# Can Immunity Induced by the Human Influenza Virus N1 Neuraminidase Provide Some Protection from Avian Influenza H5N1 Viruses?

**DOI:** 10.1371/journal.pmed.0040091

**Published:** 2007-02-13

**Authors:** Laura Gillim-Ross, Kanta Subbarao

## Abstract

Gillim-Ross and Subbarao discuss a new study in*PLoS Medicine* that suggests that immunity to the human influenza virus N1 NA cross-reacts with the avian N1 NA, and that this cross-reactivity may be sufficient to protect against infection with avian influenza virus H5N1.

## Immunity Induced by Influenza Virus Neuraminidase

The influenza virus major surface glycoproteins hemagglutinin (HA) and neuraminidase (NA) are the principal targets of the protective immune response. Licensed seasonal influenza virus vaccines are designed to elicit a protective immune response to the HA and NA proteins. However, only the concentration of HA protein is standardized in the currently approved inactivated seasonal influenza virus vaccines; the concentration of the NA protein is not. Hemagglutinin induces strain-specific neutralizing antibodies that prevent infection by antigenically related influenza viruses. Unlike HA-specific antibodies, NA-specific antibodies do not prevent influenza virus infection, and NA immunity is referred to as infection permissive [1]. However, humoral immunity induced by NA can markedly reduce virus replication and release, shortening the severity and duration of illness, a reasonable goal in the event of an influenza pandemic [2,3]. In mice, the induction of a relatively modest NA-specific humoral response is sufficient to inhibit virus replication after challenge with homologous influenza virus [4–6]. NA-specific immunity in mice provides significant cross-protection against replication of antigenically distinct viruses of the same subtype (drift variants) but not against different subtypes [7]. The degree of relatedness between the NA used for immunization and that of the challenge virus correlates well with the degree of cross-protection conferred by an NA-specific response.

Data suggest that NA antibody can modify the severity of illness.

The emergence in 1968 of an H3N2 influenza virus with a novel HA subtype (H3) provided scientists with an opportunity to evaluate the role of NA-specific antibody in protection from influenza illness in humans. In a cohort of individuals with varying levels of N2-specific antibody, the severity of clinical illness correlated with the level of NA-specific serum antibody present at the time of virus challenge (H3N2) [3]. Ten of 11 men with serum NA inhibition antibody titers less than 1:4 presented with afebrile or febrile illness following virus challenge, while one displayed no clinical signs of illness. In contrast, four of ten men with serum NA inhibition antibody titers greater than 1:4 presented with illness, while six displayed no sign of illness. These data suggest that NA antibody can modify the severity of illness associated with influenza. However, even when the NA was fully conserved between the previously circulating H2N2 virus and the newly emerged H3N2 virus, and NA immunity may have blunted the severity of the pandemic, it was not sufficient to prevent morbidity and mortality associated with the 1968 influenza pandemic.

## Does Human Influenza Virus NA-Specific Antibody Cross-React with Avian Influenza Virus NA?

Matthew Sandbulte and colleagues' new findings presented in *PLoS Medicine* provide a tantalizing suggestion that immunity to the human influenza virus N1 NA (huN1 NA) cross-reacts with the avian N1 NA (avN1 NA), and that this cross-reactivity may be sufficient to protect against infection with avian influenza virus H5N1 [8]. H1N1 influenza viruses have been circulating in the human population since 1977, and much of the population has encountered these viruses repeatedly, either through natural infection or vaccination. If huN1 NA antibodies can provide humans with cross-protection against avian influenza virus H5N1 illness, introduction of this virus into the human population may cause less devastating morbidity and mortality than has been predicted [9].

Sandbulte and colleagues vaccinated mice with two doses of DNA encoding the NA protein of A/New Caledonia/20/99 (H1N1), one of the viruses in the currently recommended trivalent seasonal human influenza virus vaccine. Antibodies to the homologous huN1 NA protein were detected in 91% of vaccinated mice, while only 13% of the mice had detectable cross-reactive antibodies to the avN1 NA protein of A/Vietnam/1203/04 (H5N1). However, the huN1 NA DNA vaccine provided a significant degree of protection from lethal challenge with A/Vietnam/1203/04 (H5N1); lethality was reduced from 100% in mock-vaccinated mice to 50% in mice vaccinated with huN1 NA, and this was accompanied by a reduction in the severity of illness as measured by weight loss. A similar level of protection was observed when sera from vaccinated mice were transferred to naïve mice, suggesting that the protection was antibody-mediated. The effect of immunization with the huN1 NA DNA vaccine on replication of A/Vietnam/1203/2004 (H5N1) in the lungs or other organs was not examined.

To determine if a similar pattern of cross-reactivity of anti-NA antibodies occurs in humans, serum samples collected from human volunteers were assayed for huN1 NA and avN1 NA antibodies by neuraminidase inhibition assay. Inhibitory activity against the huN1 NA of influenza A/New Caledonia/20/99 (H1N1) was detected in 31 of 38 sera tested, and interestingly, inhibitory activity against the avN1 NA proteins of A/Hong Kong/213/03 (H5N1) and A/Vietnam/1203/04 (H5N1) were detected in eight of 38 and nine of 38 individuals, respectively. Based on these findings, the authors hypothesize that antibodies to the huN1 NA induced by vaccines containing H1N1 influenza viruses or by natural infection with human influenza H1N1 viruses could provide humans with some degree of protection against H5N1 influenza viruses.

## What Does This Study Mean for Pandemic Influenza Preparedness Efforts?

The important question raised by this study is whether N1 NA-specific antibodies can offer some level of protection against avian influenza H5N1 viruses. Sandbulte and colleagues clearly demonstrate that vaccination of mice with DNA encoding huN1 NA induces sufficient humoral immunity to provide partial protection against H5N1 virus infection. They also provide evidence that approximately 20% of the human population have anti-NA antibodies that cross-react at low titers with the avN1 NA.

The findings of this study are very intriguing and should be investigated further, but the data are insufficient to conclude that humans with huN1 NA humoral immunity will be protected against avian influenza virus H5N1 infection. While previous studies clearly established that NA-specific antibodies can modulate the severity of influenza illness, the level of antibodies necessary to mediate such protection is not well established, and the low titer of cross-reactive NA-specific antibody detected in humans in this study may not be sufficient to protect against illness associated with avian influenza virus infection.

Additionally, although the NA was antigenically identical to that of the previously circulating H2N2 influenza virus, the 1968 H3N2 pandemic influenza virus caused severe morbidity and mortality. While NA antibodies cross-reactive with avN1 NA can be detected in the human population, the ability of these antibodies to reduce the morbidity and mortality of an H5N1 virus with an antigenically distantly related N1 NA may be minimal. It will not be easy to determine the cross-protective titer of N1 NA-specific antibodies in humans, because human infections by H5N1 viruses are sporadic.

While the current HA-based human influenza vaccines are efficacious when the epidemic strain matches the vaccine strain, consideration of the other viral proteins as targets for the development of pandemic influenza virus vaccines is prudent. We cannot predict which strain of avian influenza virus will cross the species barrier and cause a pandemic and, therefore, it is unlikely that the vaccine strains selected will exactly match the pandemic virus. In addition, avian influenza HA proteins are less immunogenic than human influenza HA proteins, requiring two doses of vaccine to induce antibody titers that correlate with protection from human influenza [10–14].

Are the data from the study by Sandbulte and colleagues sufficiently promising to recommend widespread use of human influenza vaccines to induce NA-specific antibodies in the hope that cross-reactive antibodies will protect from H5N1 infection? The concentration of NA in licensed inactivated human influenza virus vaccines is not standardized, and the correlation between this concentration and resulting titers of NA antibodies in vaccine recipients is not known. NA-specific immunity is difficult to study, because it is difficult to separate the effects of NA-specific immunity from immunity induced by the HA and other influenza virus proteins.

Some of these questions could be addressed if studies were carried out using different vaccine formulations with known concentrations of NA in study populations stratified by anti-HA antibody titers. Additionally, the ability of live attenuated influenza vaccines to induce anti-NA antibodies should be investigated, an approach adopted in a study by the late Mary Lou Clements and her colleagues [15]. One might then be able to select a vaccine that induces the highest-titer NA antibody response; the more closely related the NA of the vaccine virus and pandemic virus, the greater the potential benefit of such an approach will be. If further research supports the findings from the current *PLoS Medicine* study and widespread vaccination with human influenza virus vaccines is undertaken, the supply of embryonated eggs could become a limiting factor if efforts to immunize large populations with vaccines containing human influenza A H1N1 viruses are undertaken while H5N1 influenza vaccines are also being stockpiled. However, widespread vaccination with licensed human influenza virus vaccines could occur more rapidly than with pandemic influenza vaccines that are still under development.

Until the extent and biological benefit of cross-reactive N1 NA immunity is investigated further, it is premature to conclude that immunity induced by the human influenza virus N1 NA will provide significant protection from illness associated with avian influenza H5N1 virus infection.

## Abbreviations

HA: hemagglutinin
NA: neuraminidase
